# Cyanoacrylate glue foreign body after CT-guided localization of a pulmonary nodule during video-assisted thoracoscopic surgery: a case report

**DOI:** 10.1186/s12890-023-02321-x

**Published:** 2023-01-18

**Authors:** Jingdan Deng, Zhiwen Zeng, Yilin Liao, Haihui Zhong, Huanrong Zhang

**Affiliations:** 1 Department of Anesthesiology, Meizhou People’s Hospital, Meizhou City, 514031 Guangdong Province China; 2 Department of Thoracic Surgery, Meizhou People’s Hospital, Meizhou City, 514031 Guangdong Province China

**Keywords:** Medical glue, Airway foreign body, Localization, Small pulmonary nodules

## Abstract

**Background:**

A tracheal foreign body is a common airway aspiration that creates an emergency, which often causes unobserved respiratory problems and requires management. Iatrogenic tracheal foreign bodies are rarely observed, which results in tracheal obstruction. If the foreign body were removed from the tracheobronchial system, it would save lives. A similar case of a tracheal foreign body was focused on, which was caused by medical glue used during preoperative computed tomography localization of pulmonary nodules.

**Case presentation:**

The foreign body was deposited in the right upper bronchi, accidentally discovered after anesthesia when a double-lumen tube was located by fiber bronchoscopy. Following a video-assisted thoracoscopic surgery, the foreign body was removed using a respiratory endoscopy without subsequent adverse consequences for the patient.

**Conclusions:**

There is a risk of complications from iatrogenic airway foreign bodies for preoperative localization of pulmonary nodules by injecting cyanoacrylate glue.

## Background

Combined with recommendations for routine physical examinations to detect pulmonary nodules (PNs), the most feasible method suggested is the application of low-dose chest computed tomography (CT), which has increased significantly in detecting the presence of PNs [1]. This promotes new challenges during diagnosis; a sophisticated procedure known as video-assisted thoracoscopic surgery (VATS) has been introduced. This surgery offers a less invasive approach with minimal trauma and minor complication, leading to faster post-surgery recovery [2–4]. However, some circumstances result in VATS failure when identifying deep nodules. To overcome these clinical conditions, pre-VATS identification techniques have been introduced [5].

Although studies have reported CT-guided hook wire localization, CT-guided microcoils, and CT-guided methylene blue staining, these have shown limitations [6]. Therefore, our study focused on a cyanoacrylate glue that conferred a function similar to fibrin glue, a tissue sealant mainly used to detect wound closure and haemostasis [7]. However, limited studies describe its potential to localize the PNs. Previous research has shown the feasibility, safety, and efficiency of CT-guided cyanoacrylate localization [8]; therefore, cyanoacrylate glue after CT-guided localization of PNs will be investigated.

Coughing is a common adverse reaction of preoperative localization using medical glue. It has been reported that the incidence of cough after medical glue localization was 5–7.14% [9, 10]. It is often thought to be an airway reaction to the pungent smell of the cyanoacrylate glue, which is a type of medical sclerosing agent. Some researchers consider post-location coughing to be a mild sign of allergy [10]. As a result, it was usually not taken seriously, and no further treatment would be given. In this case, we tried to prove that coughing may also be caused by the cyanoacrylate glue as foreign bodies in the airway, and it may be an important sign of tracheal foreign bodies after the preoperative localization.

## Case presentation

**Fig. 1:**
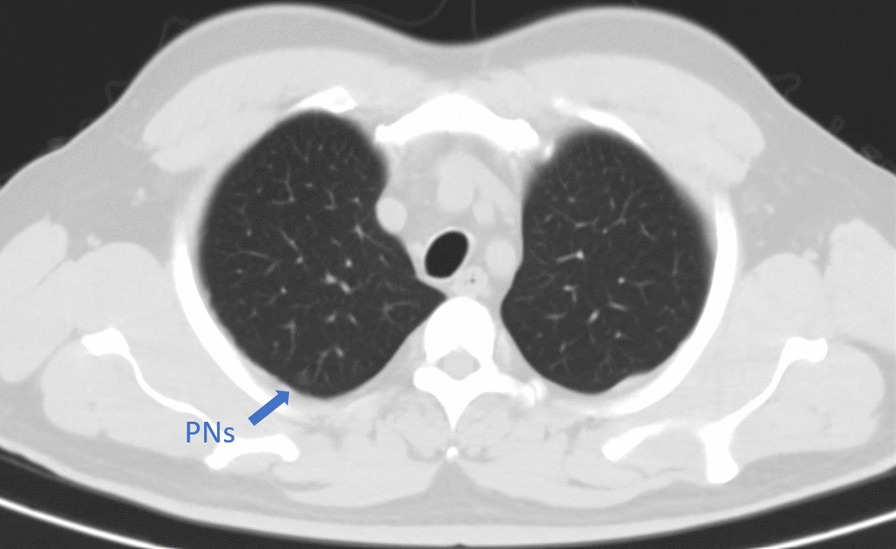
CT scan images of the targeted nodules

A 54-year-old, 78-kg male patient with a history of hypertension was admitted to the hospital complaining of PNs detected 2 months previously (Fig. 1). A VATS lung wedge resection of nodules in the right upper apical lung segment and right lower dorsal lung segments were planned. Because these two PNs were small, preoperative CT-guided localization was performed before surgery on the same day because there might be difficulty localizing the nodules intraoperatively.

**Fig. 2:**
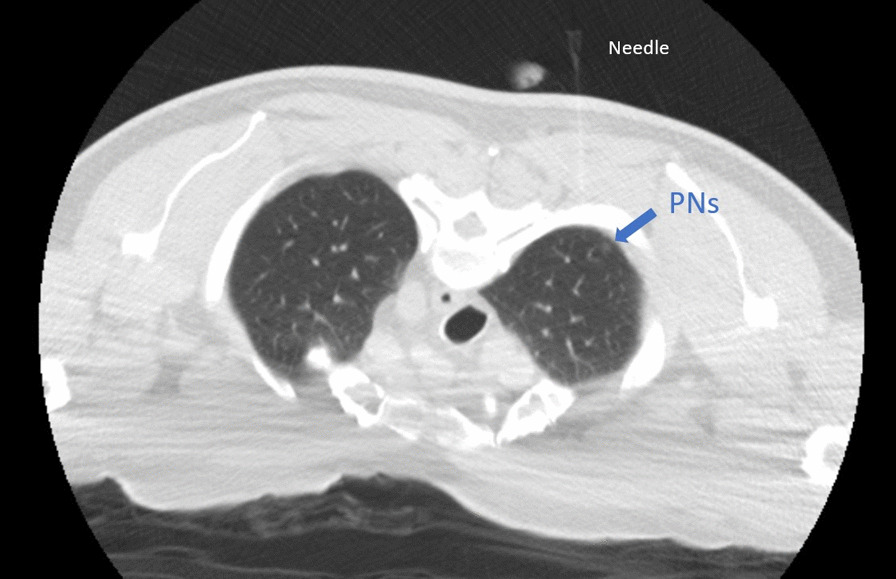
CT film of preoperative location

**Fig. 3:**
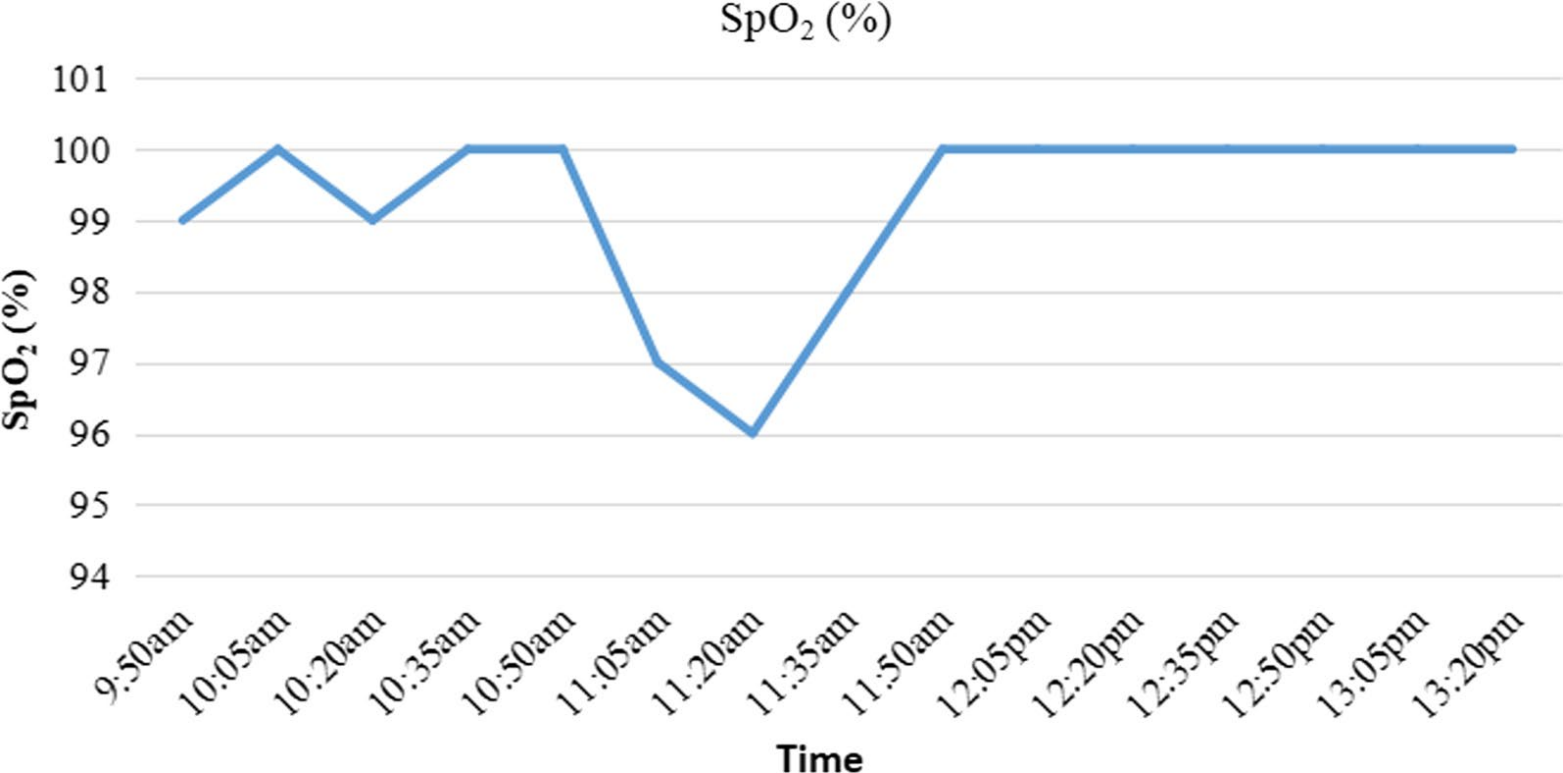
Oxygen saturation (SpO_2_) level during the surgery

The patient was placed in a prone position during localization. The procedure was guided by a multidetector (16-slice) CT helical scanner (GE lightspeed). After local anesthesia with 2% lidocaine, the 18-gauge puncture needle was inserted vertically to reach the localization site around the PNs lesions percutaneously. 0.25 ml cyanoacrylate glue (Compont^®^ medical glue) was injected at each site. One puncture was performed 0.7 cm interior into the lesion with a diameter of approximately 0.4 cm in the upper lobe of the right lung. In contrast, another puncture was performed 0.7 cm above another lesion with a diameter of approximately 0.2 cm in the lower lobe of the right lung; both used cyanoacrylate glue that was injected with an introducer needle by a radiologist (Fig. 2). After localization, the patient felt uncomfortable when coughing. The patient was admitted to the operation room after monitoring their oxygen saturation (SpO_2_) levels, maintained at 99%. Oxygen was supplied immediately, and a 37 F double-lumen left tube was intubated through their mouth after induction to obtain the right lung collapse. In addition, fiberoptic bronchoscopy was used to localize the double-lumen tube for one-lung ventilation. The anesthesiologist found that the position of the double-lumen was proper, but translucent crystallographic material was present, which was different from the secretions, such as sputum, at the bronchial opening of the right upper lobe when the bronchoscope was passed through the tracheal lumen of the tube. Combined with the coughing before anesthesia, it was thought that the crystallographic material that was present in the bronchus could be due to the preoperative CT localization. The thoracic surgeon was informed of the situation. Because the crystalline object was not observed to move in the airway under bronchoscopy and the patient's oxygenation was not affected, after a brief discussion of the patient’s condition, the surgeons and the anesthesiologist decided to perform electronic bronchoscopy immediately after thoracoscopic surgery, because the surgery did not appear to be affected. The patient underwent thoracoscopic wedge resection of the right pulmonary nodule, with a tissue margin of ≥ 2 cm, and mediastinal lymph node dissection. During intraoperative one-lung ventilation, SpO_2_ levels fluctuated between 96 and 100% (Fig. 3), and blood gas analysis showed partial pressure of oxygen (PO_2_)_,_ which was a 91.3 mm Hg fraction of inspired oxygen (FiO_2_ was 50%).

**Fig. 4:**
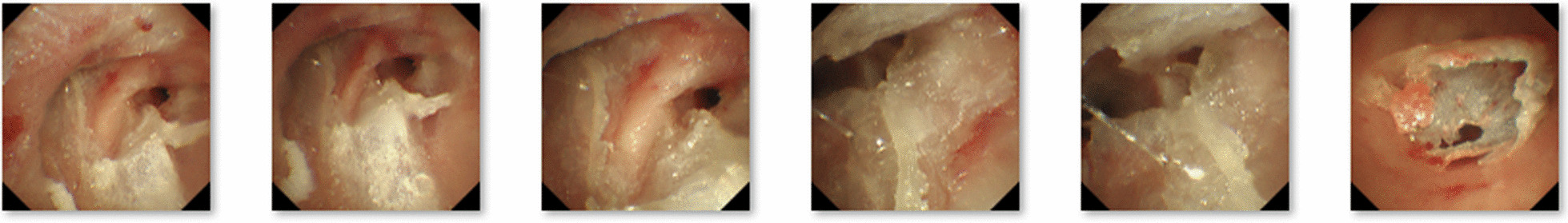
Gel-like foreign body in the right upper lobe bronchus

**Fig. 5:**
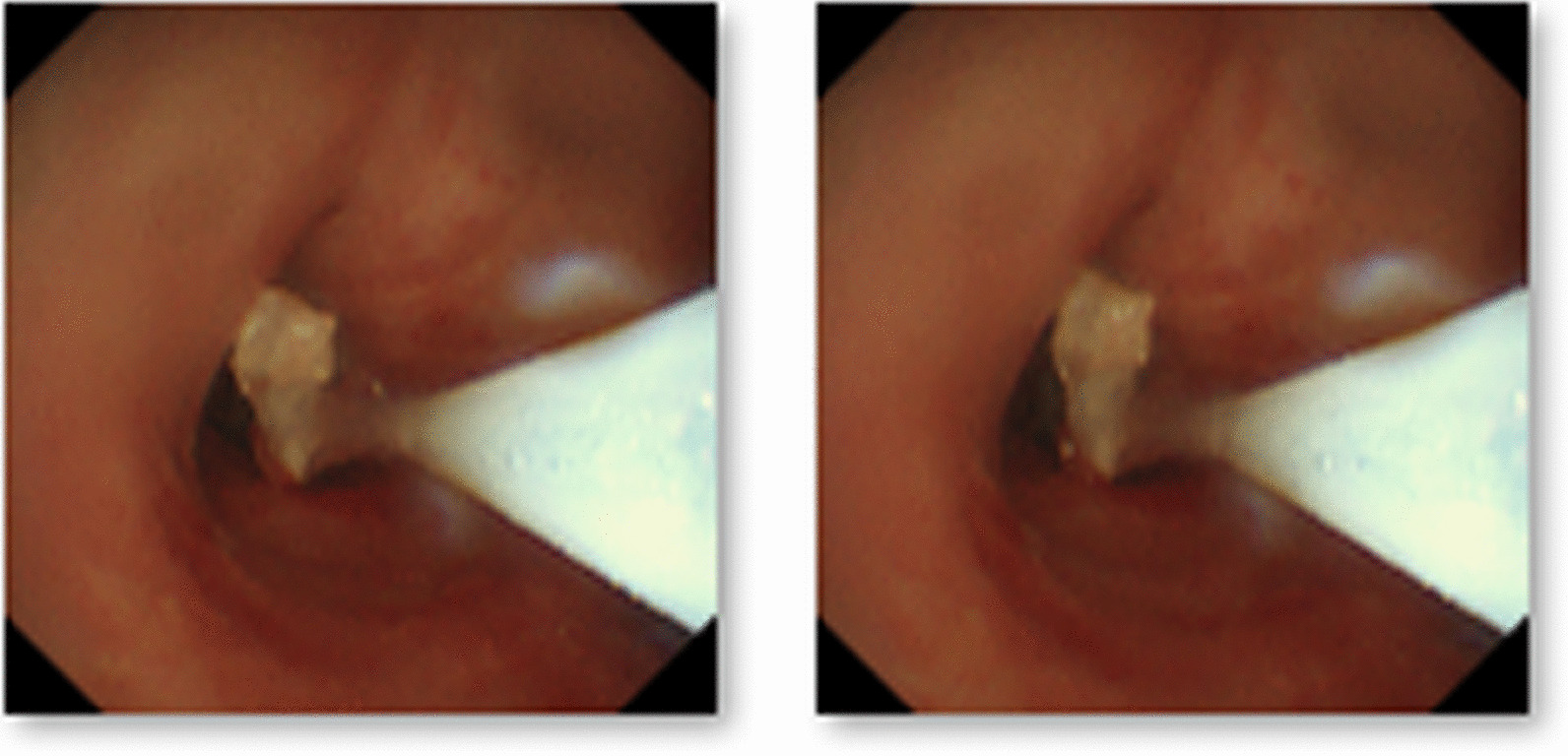
Gel-like foreign body was removed with a disposable electric trap and biopsy forceps

After thoracoscopic surgery, the patient’s position was changed from left lateral to supine. Then, the 37 F double-lumen endotracheal tube was removed, and an 8.0 ID single-lumen endotracheal catheter was inserted instead. During electronic bronchoscopy, the respiratory physician confirmed the presence of a gel-like foreign body in the right upper lobe of the bronchus. These were removed with disposable electric traps and biopsy forceps (Figs. 4 and 5). Due to the evidence of the existence of many foreign bodies, the removal process lasted for approximately 1 h until no foreign bodies were found under the electronic bronchoscope. After surgery, the patient was awake and returned to the ward after removing the tracheal tube. He had a rapid recovery without a cough, sputum, or dyspnea.

CT reports were reviewed on the first day postoperatively, and a minimum amount of air was found in the right thoracic cavity. Postoperative changes were observed in the upper lobe of the right lung. Inflammation occurred in the lower left lung and the residual right lung. Compared with the previous CT scan, the later CT scan did not diagnose ground glass nodules in the dorsal segment of the lower lobe of the right lung.

**Fig. 6:**
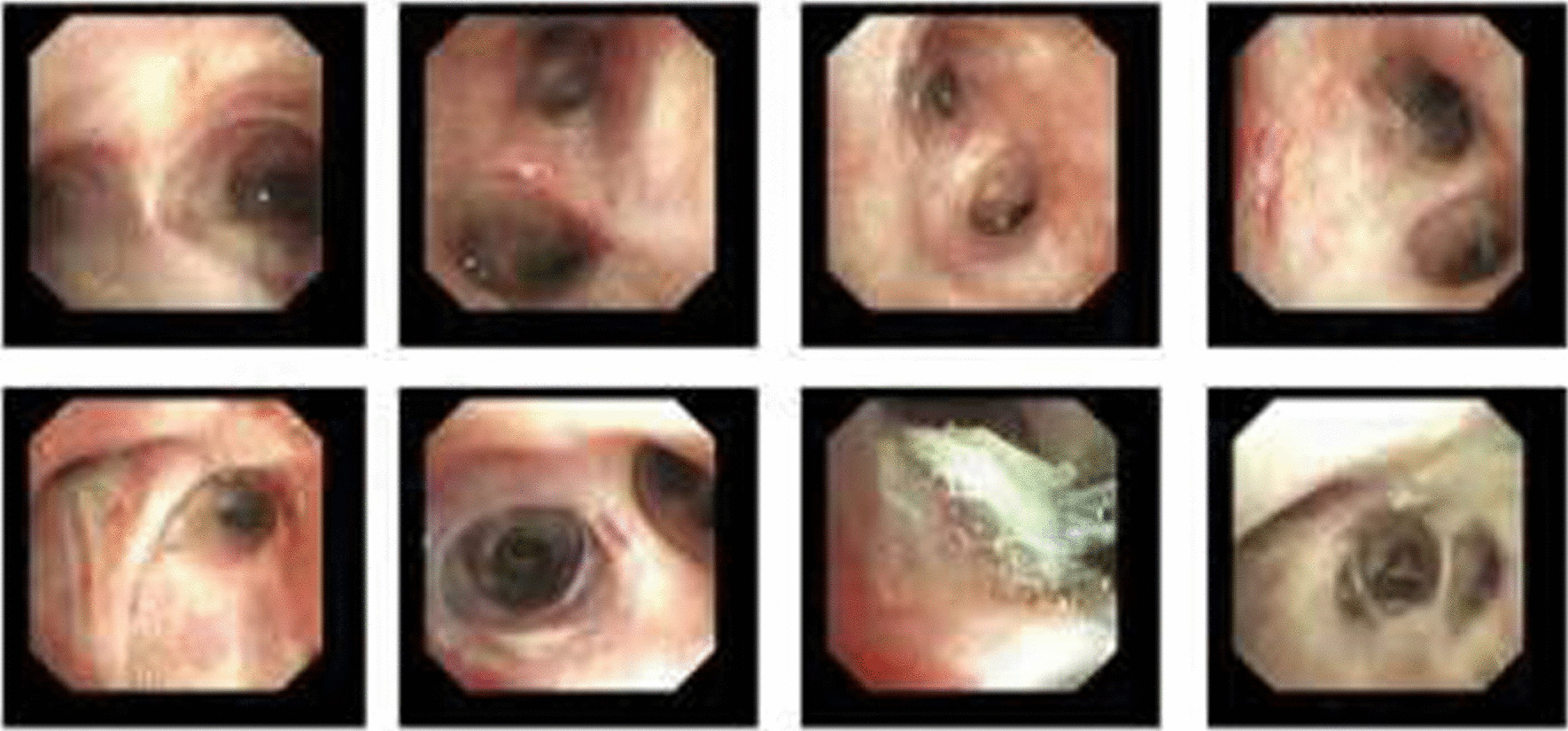
Electronic bronchoscopy re-examination on the second day postoperatively

The patient was re-examined using electronic bronchoscopy on the second day postoperatively. After endoscopic exploration of the airway under a respiratory endoscope, a gelatinous foreign body was present in the right anterior bronchus. Soon after detection, the foreign body was removed using biopsy forceps. Physiological saline lavage was performed to confirm that no foreign body residue was present (Fig. 6).

The patient was discharged successfully three days postoperatively. The pathological reports suggested a histological type of adenocarcinoma present in the lung. One month later, the patient returned to the outpatient clinic for re-examination. Clinicians thoroughly examined them using a CT scan and found no residual foreign bodies in the lung, confirming that they had recovered well without discomfort or congestion.

## Discussion and conclusions

Recently, due to the emergence of COVID-19 and the requirement of health care providers to improve people’s quality of life, it was suggested that asymptomatic individuals should undergo a chest CT scan as a part of their routine physical examinations. This practice has significantly increased the potential for the early detection of small PNs with insidious onset. VATS has become the preferred approach for the surgical management of early-stage lung cancer due to the advantages of minimally invasive procedures. However, since small PNs are challenging for finger palpation and detection by the naked eye during operations, localization and identification of intraoperative target lesions are difficult. To solve this problem, preoperative localization should be quickly considered to find the lesion. Then, surgeons can rapidly determine the exact surgical site after exposure to the surgical field.

The most used method to locate the preoperative PNs is CT-guided percutaneous puncture-assisted localization technology, bronchoscopy puncture-assisted localization technology, and CT virtual three-dimensional assisted localization technology [11]. These technologies consistently display high sensitivity with few complications [12]. Injection with medical glue is the usual CT-guided percutaneous assisted localization technique in China these years. The injection location should be < 1 cm from the target PN, and the medical glue should only be injected when no blood or gas is drawn back from the syringe. However, CT-guided percutaneous puncture-assisted positioning technology has its complications, including pneumothorax, bleeding, and cough [11]. It was demonstrated that the medical sclerosing agent had lower failure rates and complications than spring coil injection [13]. Compared with hook wire, CT-guided medical glue localization had a similar success rate, a lower risk of complications, and a longer time horizon [14].

In this case, the anesthesiologist unexpectedly found an anomaly in the airway during double-lumen positioning by a fiber bronchoscope. Combined with the preoperative situation, it was presumed that the foreign body was an iatrogenic foreign body caused by the preoperative positioning of the medical glue. The overflow of the medical glue was outside the sides of the local lung tissue excision. Because flexible fiberoptic bronchoscopes have been used to diagnose airway foreign bodies, this type of scope has a high success rate in foreign body removal [15]. Doctors decided to use electronic bronchoscopy when the patient was under general anesthesia after thoracoscopic surgery and remove the foreign body. After removal, the properties confirmed that the foreign body was medical glue. Coughing in patients after preoperative localization might be caused by the pungent smell of the medical glue [16]. In addition, the receptors of respiratory mucosa might be stimulated by mechanical and chemical stimulation, which was caused by the precipitation of a small amount of medical glue in the airway, which led to coughing.

Cyanoacrylate glue is a bio-protein glue composed of α -butyl cyanoacrylate with good biocompatibility. It is often used to stop bleeding from a wound surface, such as tissue adhesives, and to treat gastric varicose veins [17]; however, a significant risk is a vascular embolism. The application of preoperative PNs localization is another use. It is usually safe for living organisms. However, it is not easily absorbed by the tissues in a short time. If it remains in the airway, it can cause discomfort for the patient and could have some unidentified hazards [18]. A severe airway foreign body will affect gas exchange in the lungs and cause a specific inflammatory response. Here, the cause was the excessive volume of glue, or it could be due to the high pressure of the injection, which resulted in the precipitation and overflow of cyanoacrylate glue crystals into the local airway. In view of the absence of the intratracheal foreign body in another puncture location of the same patient, it is speculated that the cause may be more likely that the medical glue was injected into the small terminal bronchus by coincidence. Higher resolution CT images may be able to avoid the occurrence of erroneous injection.

Due to the careful observation of the anesthesiologist during the operation, the foreign body was quickly identified. The removal of the foreign airway body significantly reduced the patient's respiratory tract discomfort. These solutions must improve the patient's postoperative recovery and prevent the pain caused by repeated coughing caused by foreign bodies. Respiratory endoscopy was performed on day 2 postoperatively to ensure the patient's safety. This reduced the patient's concerns about their early discharge from the hospital. Injecting cyanoacrylate glue is an effective procedure for the preoperative localization of PNs; however, there is a risk of complications from iatrogenic airway foreign bodies.

## Data Availability

The datasets used and/or analyzed during the current study are available from the corresponding author on reasonable request.

## Abbreviations

CT: Computed tomography
FiO_2_: Fraction of inspired oxygen
PNs: Pulmonary nodules
PO_2_: Partial pressure of oxygen
SpO_2_: Oxygen saturation
VATS: Video-assisted thoracoscopic surgery
